# Endoscopy Management of Complete Gastric Outlet Obstruction Secondary to Elipse™ Intragastric Balloon

**DOI:** 10.7759/cureus.17542

**Published:** 2021-08-29

**Authors:** Giulio Ciprian, Jessica Khoury, Leandro Ramirez, John Miskovsky

**Affiliations:** 1 Internal Medicine, Roger Williams Medical Center, Providence, USA; 2 Department of Medicine, Roger Williams Medical Center, Providence, USA

**Keywords:** weight loss, intragastric balloon, endoscopy, stomach, gastric outlet obstruction

## Abstract

The Allurion Elipse™ device is a swallowable balloon in the form of a capsule placed without endoscopy that serves the same purpose as an intragastric balloon (IGB) used for weight loss. We report a case of a 43-year-old female who presented with a complete gastric outlet obstruction confirmed by computed tomography (CT) scan. The patient initially failed conservative management; therefore, the IGB was later removed endoscopically. This is a rare complication and one of the first cases described in the literature; therefore, further studies are needed before its widescale implementation.

## Introduction

The Allurion Elipse™ device is a swallowable balloon in the form of a capsule that is placed without endoscopy. The scope is to function as a bariatric balloon for weight loss that gradually degrades over time so that four months after placement, it deflates and then is expelled safely through the gastrointestinal tract. The balloon is usually filled with 550 mL of saline solution through a thin catheter after being swallowed [1]. Although extremely rare, this device can lead to partial or complete gastric outlet obstruction [2]. If this is the case, the device can be removed similarly to the Orbera® balloon endoscopically. However, conservative management with intravenous fluids and diet advancement is first tried before any invasive procedure is attempted [3].

## Case presentation

**Figure 1:**
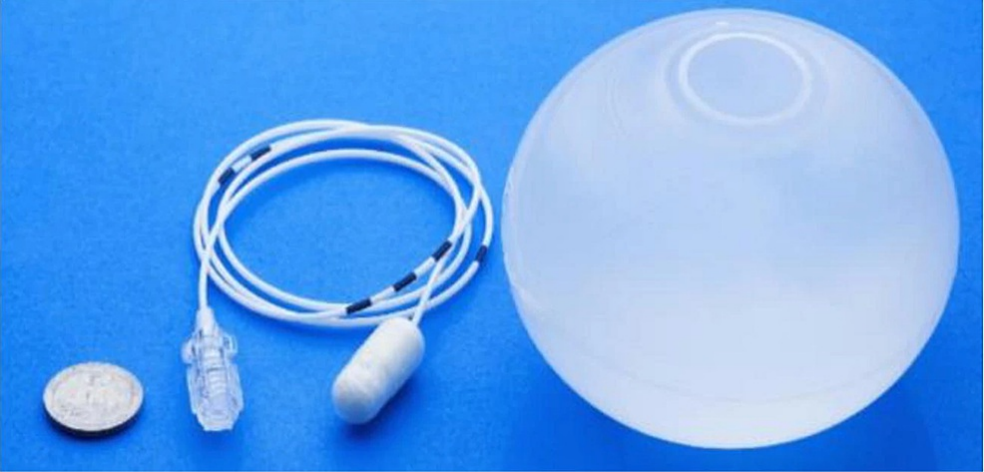
Allurion Elipse™ balloon with catheter for inflation

**Figure 2:**
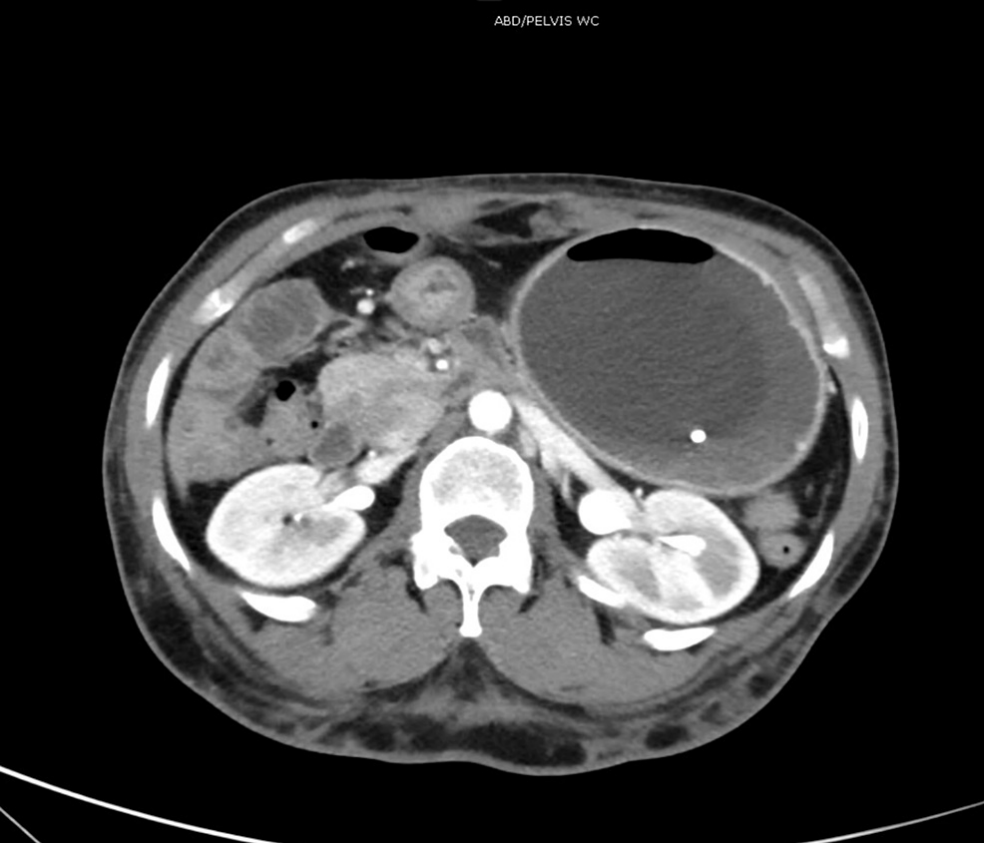
Computer tomography of abdomen showing the balloon marker

**Figure 3:**
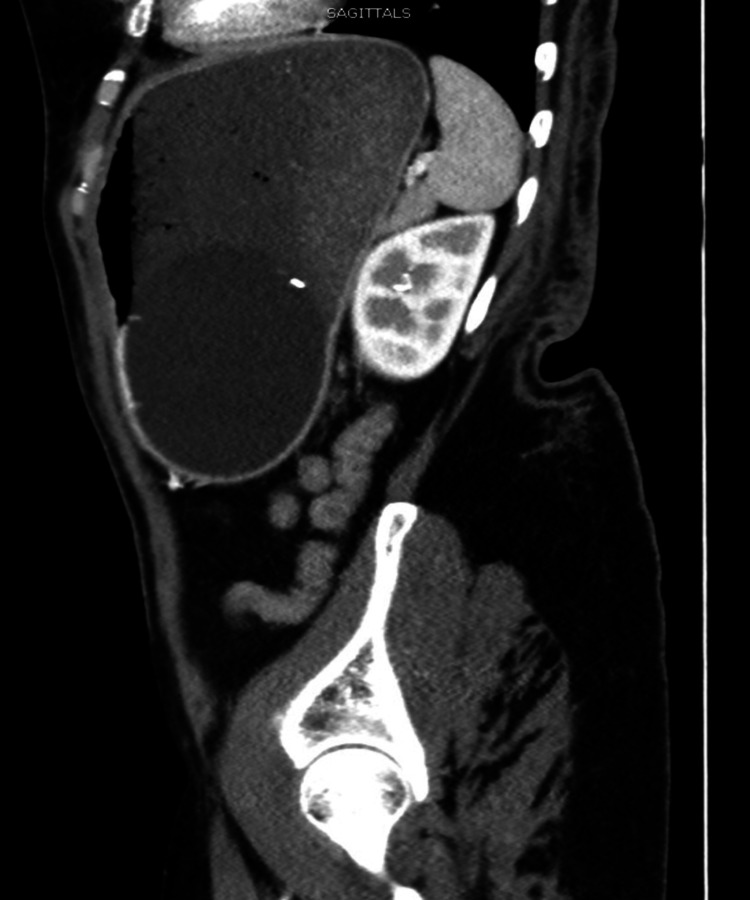
Sagittal view of the Elipse™ intragastric balloon obstructing the gastric outlet

**Figure 4:**
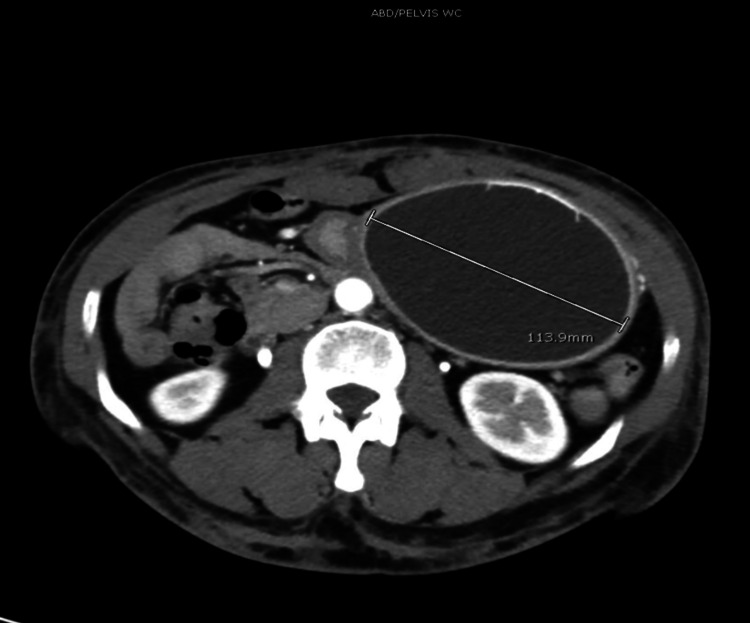
Computer tomography of abdomen showing balloon placed in the stomach and its diameter

**Figure 5:**
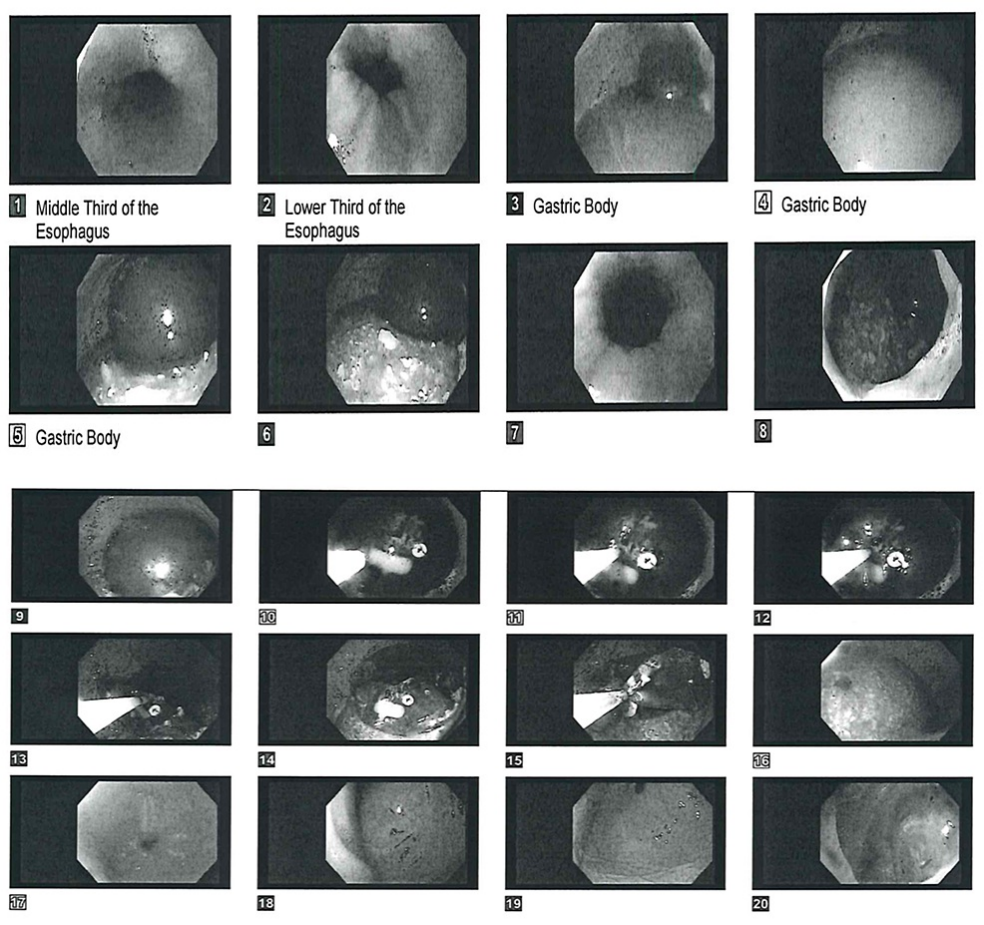
Endoscopy showing retrieval of the Elipse™ balloon and esophagogastroduodenoscopy after its removal

**Figure 6:**
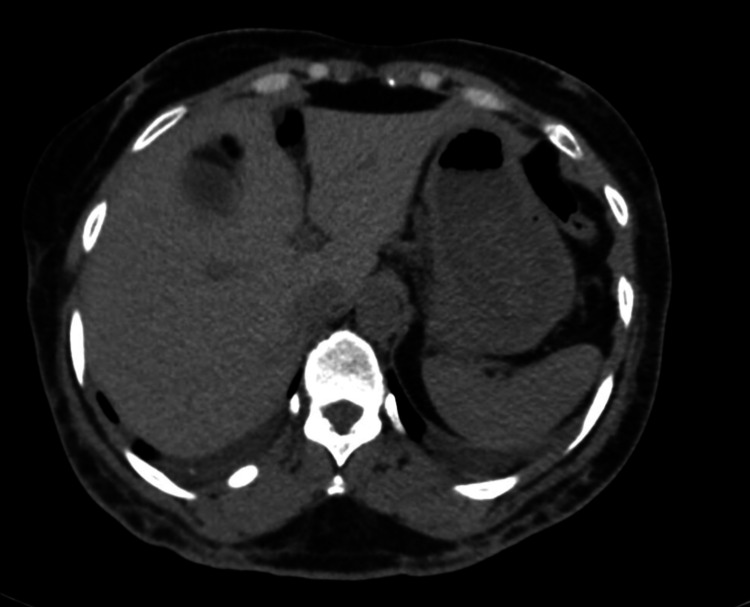
Computer tomography of abdomen showing resolution of the obstruction after endoscopic retrieval of the balloon

A forty-three-year-old female with no significant medical history but status post abdominoplasty and breast enhancement surgery presents to the hospital with ongoing abdominal pain for three weeks. The patient reported to have had a bariatric surgical procedure in the Dominican Republic consisting of an Allurion Elipse™ balloon placed into her stomach (Figure 1). The device was confirmed to be positioned correctly by abdominal X-ray without signs of hyperinflation. From day one post-surgery, she endorsed nausea, abdominal discomfort, and vomiting and was not able to tolerate p.o. solid and minimal p.o. liquids. She reported the emesis to be non-bilious in nature, with very scanty blood secretions at the end of the vomitus. Moreover, she reported occasional diarrhea described as clear-green in nature without any foul smell associated with it. Since the placement of the device, the patient reports losing about 25 pounds. On admission, the patient was normotensive and afebrile without respiratory distress. Her abdominal pain was managed with morphine and proton pump inhibitors, nausea with Zofran™. An abdominal computer tomography (CT) performed on arrival revealed a distended stomach with complete obstruction of the gastric outlet (Figures 2-3). Furthermore, a gastric bariatric balloon measuring up to 11.6 cm in size was visualized with overall gastric distension measuring up to 11 cm (Figure 4). The patient was initially placed n.p.o. and electrolytes were repleted intravenously; however, the patient did not tolerate the advancement of the diet to clear liquids. The gastroenterology team and the inventor of the device were consulted, both of which initially recommended symptomatic and conservative management and to observe for any improvement. However, on the fifth day of hospitalization, a decision to remove the balloon endoscopically was made since no significant improvements could be observed. During endoscopy, an intragastric balloon compatible with the Elipse™ was noticed with no signs of hyperinflation and fully intact in a proper position. Copious fluid was suctioned with some solid debris just underneath the fluid. Using the Orbera® removal system, the balloon was punctured, and approximately 700cc of clear fluid was aspirated. Thereafter, the balloon was retrieved successfully with rat-tooth forceps and found completely intact upon inspection (Figure 5). An esophagogastroduodenoscopy (EGD) was then performed to ensure no injury in the esophagus or cardias sphincter was present, both of which appeared normal. Pylorus and duodenum also appeared normal. After the procedure, the patient felt better and was able to tolerate diet advancement. Repeat CT scan showed complete resolution of the obstruction (Figure 6). The patient was discharged home without any complications.

## Discussion

At the moment, the device Allurion Elipse™ is not yet FDA approved for use in the United States. While some health authorities outside of the US have a more liberal approach, it is paramount that more studies and research are performed on this device before its introduction into the North American market. While its way of being deployed and extracted without undergoing anesthesia or endoscopy seems appealing for many patients and providers, more studies are needed for definitive recommendations on the potential carcinogenic risk deriving from the device being in direct contact with the digestive tract lining even after deflation [4-6]. Furthermore, its risks associated with hyperinflation causing complete gastric obstruction in a similar way to pyloric stenosis raise the need for more in-depth trials and safety protocols. Moreover, there has been a case of distal gastric migration, which can raise concerns for rupture and complications derived from it, such as compression of nearby anatomical structures, pancreatitis, and small bowel obstruction [7].

## Conclusions

Although initial data showed that the Elipse™ intragastric balloon to be safe overall, serious complications can still occur. As medical providers, we are not able to release definitive recommendations on the safety of the Allurion Elipse™ balloon until further studies and trials are available in the literature.
